# The Role of RelA and SpoT on ppGpp Production, Stress Response, Growth Regulation, and Pathogenicity in Xanthomonas campestris pv. *campestris*

**DOI:** 10.1128/spectrum.02057-21

**Published:** 2021-12-22

**Authors:** Kaihong Bai, Huayu Yan, Xing Chen, Qingyang Lyu, Na Jiang, Jianqiang Li, Laixin Luo

**Affiliations:** a Department of Plant Pathology, China Agricultural University, Beijing Key Laboratory of Seed Disease Testing and Control, MOA Key Lab of Pest Monitoring and Green Management, Beijing, People’s Republic of China; b CAS Key Laboratory of Environmental and Applied Microbiology, Chengdu Institute of Biology, Chinese Academy of Sciences, Chengdu, People’s Republic of China; USDA - San Joaquin Valley Agricultural Sciences Center

**Keywords:** ppGpp, *Xanthomonas campestris* pv. *campestris*, stress resistance, viable but nonculturable, pathogenicity

## Abstract

The alarmone ppGpp plays an important role in the survival of bacteria by triggering the stringent response when exposed to environmental stress. Although Xanthomonas campestris pv. *campestris* (Xcc), which causes black rot disease in crucifers, is a representative species of Gram-negative phytopathogenic bacteria, relatively little is known regarding the factors influencing the stringent response in this species. However, previous studies in other Gram-negative bacteria have indicated that RelA and SpoT play a critical role in ppGpp synthesis. The current study found that these proteins also had an important role in Xcc, with a Δ*relA*Δ*spoT* double mutant being unable to produce ppGpp, resulting in changes to phenotype including reduced production of exopolysaccharides (EPS), exoenzymes, and biofilm, as well the loss of swarming motility and pathogenicity. The ppGpp-deficient mutant also exhibited greater sensitivity to environment stress, being almost incapable of growth on modified minimal medium (mMM) and having a much greater propensity to enter the viable but nonculturable (VBNC) state in response to oligotrophic conditions (0.85% NaCl). These findings much advance our understanding of the role of ppGpp in the biology of Xcc and could have important implications for more effective management of this important pathogen.

**IMPORTANCE** Xanthomonas campestris pv. *campestris* (Xcc) is a typical seedborne phytopathogenic bacterium that causes large economic losses worldwide, and this is the first original research article to investigate the role of ppGpp in this important species. Here, we revealed the function of RelA and SpoT in ppGpp production, physiology, pathogenicity, and stress resistance in Xcc. Most intriguingly, we found that ppGpp levels and downstream ppGpp-dependent phenotypes were mediated predominantly by SpoT, with RelA having only a supplementary role. Taken together, the results of the current study provide new insight into the role of ppGpp in the biology of Xcc, which could also have important implications for the role of ppGpp in the survival and pathogenicity of other pathogenic bacteria.

## INTRODUCTION

The alarmone (p)ppGpp, a global regulator in bacteria, was first reported in Escherichia coli in 1969 (1). Subsequent research has shown that ppGpp is an important global regulator in Gram-negative bacteria that influences many biological processes, in particular triggering the stringent response that allows bacteria to survive unfavorable environmental conditions, especially nutrient limitation (2, 3). For example, in E. coli, the absence of amino acids leads to the stalling of ribosomes and the binding of uncharged tRNA molecules to the ribosomal A site, which activates the RelA protein to synthesize ppGpp (4, 5). However, in the absence of other nutrients, such as phosphate, iron, and fatty acids, biosynthesis of ppGpp is achieved by an alternative mechanism involving the bifunctional enzyme SpoT, which contains both ppGpp synthetase and hydrolase domains (4). After synthesis, ppGpp can influence cellular processes via transcription and translation. In the former case, ppGpp can have an inhibitory effect by binding the RNA polymerase directly in combination with DksA in order to prevent the transcription of rRNA promoters and stimulate the transcription initiation of amino acid biosynthesis promoters or by a second method by which it alters the utilization of sigma factors to induce the global changes in transcription initiation (4). In the latter case, ppGpp inhibits the initiation and extension of translation by binding to the EF-Tu, EF-G, and IF2 translation factors. As well as its effects on replication and nutrient metabolism (4, 6), recent studies have indicated that ppGpp could have an important role in the virulence of plant-pathogenic bacteria, including Pectobacterium atrosepticum (previous synonym: Erwinia carotovora subsp. *atroseptica*), Pseudomonas syringae, Xanthomonas citri subsp. *citri*, and Erwinia amylovora (7–11). For example, it was found that ppGpp-deficient mutants of P. syringae exhibited significantly reduced pathogenicity and downregulation of virulence-related genes, such as the type III secretion system. In X. citri subsp. *citri*, the ppGpp-deficient mutant showed reduced symptom development, which was confirmed by decreased gene expression levels of type 3 secretion system (T3SS), type 2 secretion system (T2SS), and other genes related to virulence from transcriptome sequencing (RNA-Seq) data (11). Similarly, ppGpp has been observed to mediate virulence in several other pathogens, including exoenzyme production in Pe. atrosepticum, biofilm formation in Mycobacterium smegmatis, exopolysaccharides (EPS) secretion in Ps. syringae, and swarming motility in Agrobacterium tumefaciens (8, 12–15).

The enzymes responsible for ppGpp metabolism in bacteria can be divided into three main categories: long RelA/SpoT homologue (RSH) proteins, which contain both a synthetase and hydrolase domain, small alarmone synthetases (SAS), which contain only a synthetase domain, and small alarmone hydrolases (SAH), which contain only a hydrolase domain (16), although only the RSH and SAS enzymes can catalyze the key step in ppGpp synthesis transferring the phosphate group from ATP to 3′ hydroxide radical of GDP (17). Evidence to date suggests that some Gram-negative bacteria in *Beta*- and *Gammaproteobacteria* generally utilize two homologous RSH enzymes, RelA and SpoT, and possibly contain SAS to synthesize (p)ppGpp (such as fpRel in *Ps. syringae* pv. *tomato* and RelV in Vibrio cholerae) (9, 16, 18), while Gram-positive bacteria, including Bacillus subtilis, Streptococcus mutans, Staphylococcus aureus, Enterococcus faecalis, and Corynebacterium glutamicum, contain only a bifunctional RSH protein in combination with SASs, termed RelP (SAS2, YwaC) and RelQ (SAS1, YjbM) (19–23).

Xanthomonas campestris pv. *campestris* (Xcc), a representative species of Gram-negative plant-pathogenic bacteria, is an important seed-borne pathogen (24–26) that causes black rot disease of crucifers, which is the leading cause of economic loss to these crops worldwide. In the absence of reliable resistant crop varieties or chemical interventions, the management of black rot relies heavily on seed treatment and the planting of certified pathogen-free seeds. However, this strategy has been complicated by the discovery that Xcc can enter the viable but nonculturable (VBNC) state after exposure to abiotic stress such as copper, sterile soils, and oligotrophic conditions (27). The VBNC state was first reported in E. coli and V. cholerae (28) and is considered a resistance response that allows predominantly Gram-negative species and some non-spore-forming Gram-positive species to survive periods of unfavorable environmental conditions. Although much of the research regarding the VBNC state has focused on human pathogens, the VBNC state has also been observed in many plant pathogens, including A. tumefaciens, Ralstonia solanacearum, Er. amylovora, Xanthomonas axonopodis pv. *citri*, Ps. syringae pv. *syringae* and Ps. syringae pv. *tabaci*, Xylella fastidiosa, Clavibacter michiganensis, and Acidovorax citrulli (29–36). This presents a significant problem for the seed certification strategy used to manage soilborne pathogens such as Xcc, as conventional culturability-based techniques are unable to detect contamination with VBNC cells, which can later resuscitate under favorable conditions and act as a primary source of inoculum (37). Consequently, it is critical that the factors leading to the induction of the VBNC, including the role of ppGpp, are better understood, and it is therefore interesting to note that recent studies in E. coli have indicated that *relA* and *spoT* were upregulated during the VBNC state and that ppGpp-deficient mutants lost culturability more rapidly than the wild type (38, 39).

The current study was initiated to investigate the hypothesis that ppGpp also plays a key role in both the stringent response and induction and maintenance of the VBNC state in Xcc. Having established that Xcc could be reproducibly induced into the VBNC state by CuSO_4_ treatment and resuscitated by the addition of Luria-Bertani (LB) broth, tryptic soy broth (TSB), amino acid, and sodium pyruvate in preliminary experiments, the current study went on to examine the role of RelA and SpoT in ppGpp production and its effect on stress resistance, biofilm production, and exoenzyme secretion, as well as its effect on pathogenicity and the VBNC state in a series of RelA/SpoT mutants and complementation strains. To the best of our knowledge, this constitutes the first report regarding the role of ppGpp in Xanthomonas campestris pv. *campestris* and provides important insight into the role of ppGpp in the VBNC state and survival of this important plant-pathogenic bacterium.

## RESULTS

### The *relA*/*spoT* system of Xanthomonas campestris pv. *campestris* 8004.

Bioinformatic analysis of the Xcc 8004 genome identified two putative RSH proteins, XC_RS05900 and XC_RS04795, which corresponded to RelA and SpoT, respectively. Further analysis indicated that both proteins contained the ppGpp synthetase domain, as well as other characteristic motifs, such as a hydrolase domain (Fig. S1). The ppGpp synthetase domains of the Xcc RelA and SpoT proteins were then used to search for further homologous proteins in the Xcc 8004 genome. Although nine possible candidates were identified, none exhibited significant homology (Table S1) or contained the characteristic synthetase domain.

### The effect of *relA* and *spoT* on growth, EPS production, biofilm formation, extracellular enzymes secretion, and swarming motility.

A series of deletion mutants and complementation strain were developed to study the influence of the *relA* and *spoT* genes on the stringent response, VBNC state, and pathogenicity of Xcc. Although Δ*relA* and Δ*relA*Δ*spoT* mutants were successfully generated using the triparental mating method, attempts to produce a single gene knockout of the *spoT* gene were unsuccessful. Similar results were found in a previous study of E. coli (17), which suggests that SpoT could play a critical role in the regulation of ppGpp accumulation via its hydrolase activity. It can be inferred that absence of SpoT results in death of the Δ*spoT* transformants because the ppGpp produced by RelA accumulates to lethal levels.

The expression levels of *relA* and *spoT* in the Xcc mutants and complementation isolates were investigated by quantitative PCR (qPCR) compared with those in the wild type, which confirmed that neither the Δ*relA* nor the Δ*relA*Δ*spoT* mutant had any detectable *relA* expression (Fig. S2A) and that the Δ*relA*Δ*spoT* mutant had no *spoT* expression (Fig. S2B). The qPCR also confirmed that *relA* expression could be restored to the deletion mutants by the reintroduction of a functional *relA* gene in the complementation strains, and it was interesting to note that *relA* expression in the complementation strains was significantly higher than that of the wild-type isolate and that expression in the Δ*relA*Δ*spoT*(*relA*) complementation strain was higher than that of Δ*relA*(*relA*) (Fig. S2A). Similar results were observed in the Δ*relA*Δ*spoT*(*spoT*) mutant, which had significantly higher levels of *spoT* expression compared to either the wild-type isolate or the Δ*relA*(*relA*) complementation strain, which itself had slightly (though not significantly) increased levels of *spoT* expression (Fig. S2B). These results indicated that both the complementation genes were under the control of their native promoters but do seem consistent with the hypothesis that SpoT has a more important role in the regulation of ppGpp accumulation than RelA. The lack of any detectable *relA* or *spoT* expression in the Δ*relA*Δ*spoT* mutant was also evidence that this strain lacked the capacity to produce ppGpp, a fact that was later confirmed in the response to stress experiments described below.

**FIG 1:**
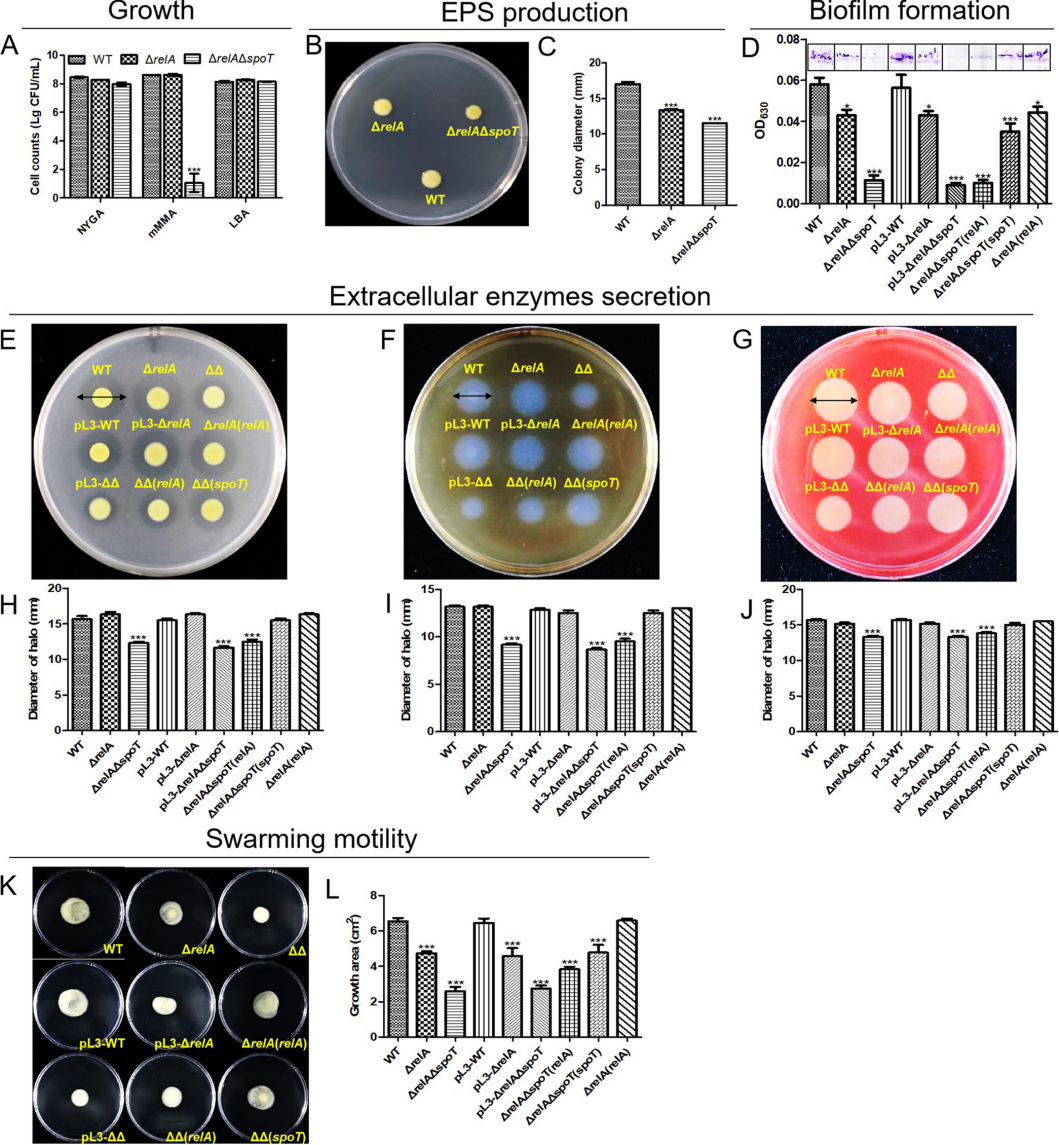
Effect of *relA* and *spoT* on the bacterial growth, EPS production, biofilm formation, secretion of extracellular enzymes, and swarming motility in Xanthomonas campestris pv. *campestris*. The top left panel (A) depicts the bacterial titers data collected from colonies after 4 days of growth on the NYGA and LBA or after 5 days of growth on modified minimal media (mMM). The top middle panel shows the colonies produced during the exopolysaccharides (EPS) secretion assay after 4 days of culture on NYGA containing 2% glucose (B) and the colony diameter data from the EPS assay (C). Bars correspond to one standard deviation (SD) from the mean (*n* = 3), while asterisks indicate significant differences (*P* < 0.001) calculated from a two-way ANOVA in comparison to the WT sample. The top right panel (D) depicts the glass-bound biofilm stained with crystal violet, while the bar graph below shows the corresponding absorbance data (630 nm) when the biofilm was dissolved in absolute ethanol. The middle panels depict the zones of digestion associated with protease activity (E), amylase activity (F), and cellulase activity (G) on NYGA amended with1% (wt/vol) skimmed milk, 0.1 (wt/vol) soluble starch, and 0.5% (wt/vol) carboxymethyl cellulose, respectively, while the bar graphs below (H to J) show the corresponding zone of activity area data. The bottom panel on the left (K) depicts the bacterial growth area after 4 days of culture on semisolid NYGA media, while the bar graph on the right (L) shows the corresponding statistical data of bacterial growth area for different strains. WT, Δ*relA*, and Δ*relA*Δ*spoT* (ΔΔ) indicate the wild type, single mutant, and double mutant, respectively, while the pL3-WT indicates control strain, which harbors an empty vector, used during complementation, and the suffixes (*relA*) and (*spoT*) indicate complementation with functional copies of *relA* and *spoT*, respectively. Bars indicate one standard deviation (SD) form the mean (*n* = 3), while asterisks indicate significant differences (*P* < 0.05) derived from a one-way ANOVA in conjunction with Dunnett’s multiple-comparison test.

The growth of the wild type and the Δ*relA* and Δ*relA*Δ*spoT* (ppGpp-deficient) mutants differed when they were grown on the NYGA, mMMA, LBA media (Fig. 1A). Examination of the bacterial titers from individual colonies indicated that the colony of the Δ*relA*Δ*spoT* mutant was barely formed on mMMA, resulting in a massive reduction in the number of CFU, only 18.25 ± 16.82 CFU/mL compared to (4.10 ± 0.14) × 10^8^ CFU/mL and (4.10 ± 0.77) × 10^8^ CFU/mL in the wild type and Δ*relA* mutant, respectively. However, no differences were found between the cell titers of Δ*relA*Δ*spoT* mutant on LBA and NYGA ([1.40 ± 0.05] ×10^8^ and [9.55 ± 0.26] × 10^7^ CFU/mL, respectively), which indicates that ppGpp deficiency restricts the capacity of Xcc to survive under nutrient-limiting conditions. Further investigation revealed that Δ*relA*Δ*spoT* as well as the single gene Δ*relA* mutant also produced smaller colonies when grown on NYGA plates amended with 2% glucose, a medium used as an indicator of EPS production, with colony diameters of 11.5 ± 0.0 and 13.3 ± 0.3 mm, respectively, compared to 17.0 ± 0.5 mm for the wild type (Fig. 1B and C). Similar results were also found with regard to the biofilm formation. In this complex interaction, the amount of biofilm was significantly reduced in Δ*relA* and especially Δ*relA*Δ*spoT*, with the biofilm/crystal violet extract producing lower optical density at 630 nm (OD_630_) values of 0.043 ± 0.005 and 0.011 ± 0.004, respectively, compared to 0.058 ± 0.006 in the wild type (Fig. 1D). However, in this case, complementation with functional *relA* and *spoT* genes was insufficient to restore the wild-type phenotype, with the introduction of *relA* having no effect on Δ*relA*(*relA*) or Δ*relA*Δ*spoT*(*relA*), which had OD_630_ values of 0.043 ± 0.003 and 0.010 ± 0.003, respectively, and the functional *spoT* gene only partially restoring phenotype in the Δ*relA*Δ*spoT*(*spoT*) complementation strain (OD_630_ = 0.035 ± 0.007). In this case, the secretion of extracellular protease, amylase, and cellulase was greatly reduced in the Δ*relA*Δ*spoT* mutant, resulting in a reduction in the zone of activity from 15.67 ± 0.76, 13.17 ± 0.29, and 15.67 ± 0.29 mm in the wild type to 12.33 ± 0.29, 9.17 ± 0.29, and 13.33 ± 0.29 mm, respectively, in Δ*relA*Δ*spoT* (Fig. 1E to J). It was also extremely interesting to note that although the Δ*relA* mutant did not differ significantly compared to the wild type, the introduction of a functional *spoT* gene completely restored the wild-type phenotype in the Δ*relA*Δ*spoT*(*spoT*) complementation strain, indicating that enzyme secretion was largely mediated by the activity of SpoT. Taken together, these results indicate that ppGpp plays an important role in the response of Xcc to oligotrophic conditions, EPS production, biofilm formation, and secretion of extracellular enzymes.

The swarming motility of the deletion mutants was also found to be significantly altered, but in this case the effect was more clearly defined, with the phenotype seeming to be mediated by both RelA and SpoT. Similar to the EPS, biofilm, and extracellular enzymes assays, swarming motility was reduced in both mutants but to a higher degree in the double mutant, which almost completely lacked the ability to swarm on semisolid NYGA media (Fig. 1K). This resulted in much smaller growth areas of 4.74 ± 0.20 and 2.59 ± 0.42 cm^2^ in Δ*relA* and Δ*relA*Δ*spoT*, respectively, compared to 6.55 ± 0.30 cm^2^ in the wild type (Fig. 1L). However, while the introduction of functional genes completely restored phenotype to the level of the wild type and Δ*relA* mutant in the complementation strains Δ*relA*(*relA*) (6.58 ± 0.21 cm^2^) and Δ*relA*Δ*spoT*(*spoT*) (4.78 ± 0.75 cm^2^), respectively, it was noted that *relA* could also partially restore phenotype in the complementation strain Δ*relA*Δ*spoT*(*relA*) (3.84 ± 0.25 cm^2^). This result indicates that, while the swarming motility phenotype is predominantly mediated by SpoT, it can also be influenced by RelA.

Taken together, these results indicate that the interaction between RelA and SpoT and their effect on ppGpp production and downstream biological processes in Xcc could be more complicated than previously thought.

### The effect of RelA, SpoT, and ppGpp on pathogenicity in Xanthomonas campestris pv. *campestris* 8004.

**FIG 2:**
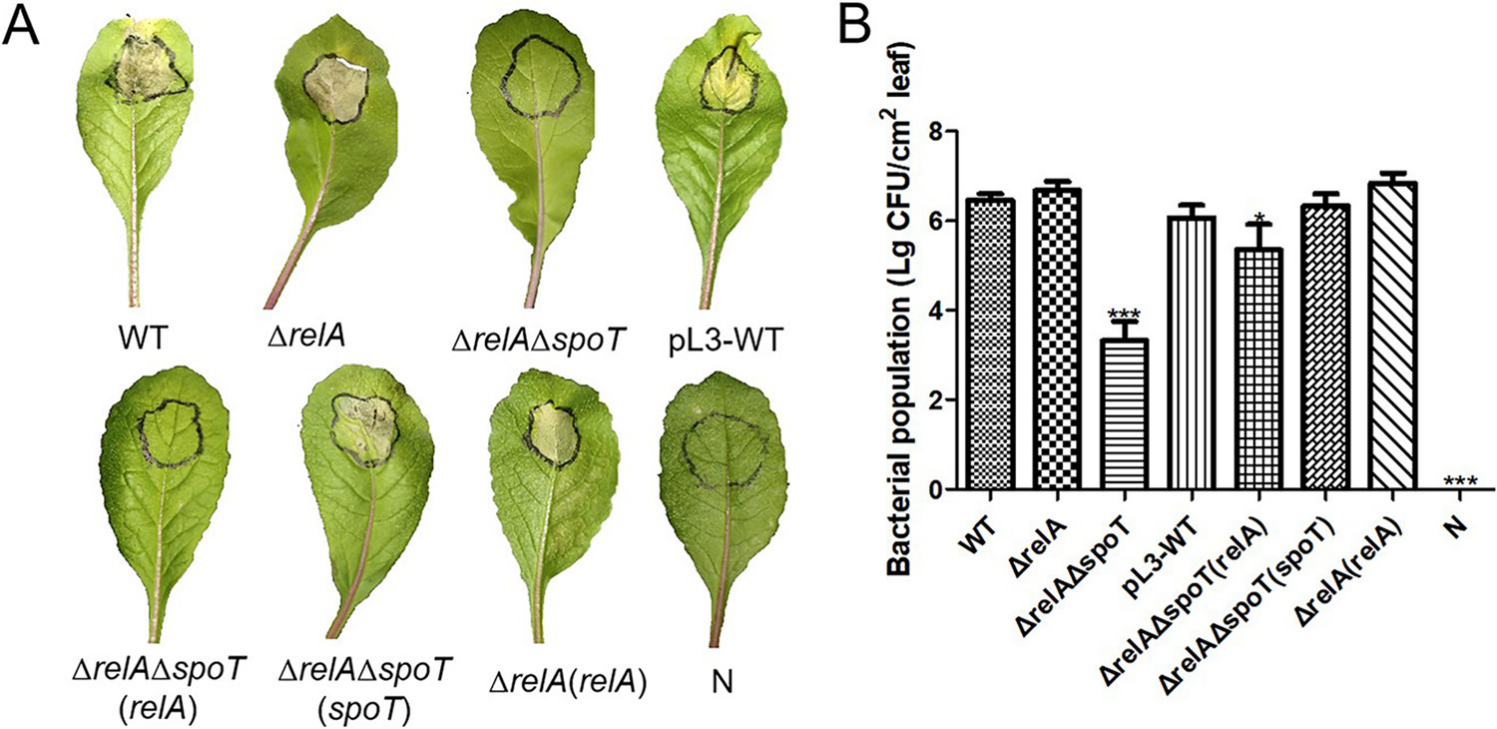
Effect of *relA* and *spoT* on the pathogenicity of Xanthomonas campestris pv. *campestris*. The left panel (A) depicts the visual symptoms of disease on radish (Raphanus sativus L. cv. Japan 501) leaves injected with bacterial suspensions observed at 7 days postinoculation (dpi). Note that typical chlorotic and necrotic lesions can be seen on all of the leaves with the exception of those that were inoculated with SpoT-deficient strains. The right graph (B) shows the bacterial titers reisolated from inoculated radish leaves at 7 days postinoculation (dpi) and cultured to obtain quantitative colonies for each strain. N, WT, Δ*relA*, and Δ*relA*Δ*spoT* indicate the negative control (0.85% NaCl), wild type, single mutant, and double mutant, respectively, while the prefix pL3- indicates control strain used during complementation and the suffixes *relA* and *spoT* indicate complementation with functional copies of *relA* and *spoT*, respectively. Bars indicate one standard deviation (SD) form the mean (*n* = 3), while asterisks indicate significant differences (*P* < 0.05) derived from a one-way ANOVA in conjunction with Dunnett’s multiple-comparison test.

The pathogenicity of the deletion mutants, complementation strains, and wild-type isolate was assessed by injecting bacterial suspensions into the leaves of the red radish (Raphanus sativus L.). Visual inspection at 7 days postinfection (dpi) revealed that all strains, with the exception of the SpoT-deficient strains [Δ*relA*Δ*spoT* and Δ*relA*Δ*spoT*(*relA*)], caused typical chlorotic and necrotic symptoms around the site of injection (Fig. 2A).

However, bacterial reisolation indicated that the pattern of infection was slightly more complex than first appeared, as the data indicated that the introduction of a functional *relA* gene to the Δ*relA*Δ*spoT* mutant might facilitate some degree of infection even though there were no visible signs of disease, as the Δ*relA*Δ*spoT*(*relA*) complementation strain yielded a bacterial titer of 2.04 × 10^6^ CFU/cm^2^, which was intermediate between the values for the Δ*relA*Δ*spoT* mutant (2.12 × 10^4^ CFU/cm^2^) and the wild-type isolate (6.51 × 10^6^ CFU/cm^2^) (Fig. 2B). Taken together, these results confirmed that ppGpp plays a key role in the pathogenicity of Xcc but that the expression of *spoT* is of much greater importance than that of *relA*.

### The effect of environmental stress on RelA/SpoT expression and ppGpp accumulation.

**FIG 3:**
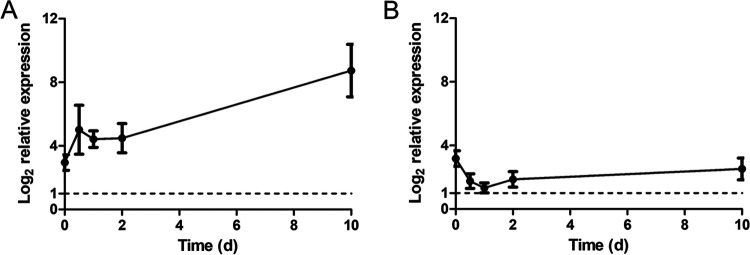
Relative expression of *relA* and *spoT* in Xanthomonas campestris pv. *campestris* exposed to copper stress. The relative expression of *relA* (A) and *spoT* (B) in Xcc 8004 samples with an initial OD_600_ of 0.18 were recorded at 5 min, 0.5 d, 1 d, 2 d, and 10 d compared with 0 min after exposure to 50 μM Cu^2+^. Bars correspond to one standard deviation (SD) from the mean (*n* = 3).

Preliminary studies indicated that the VBNC state could routinely be induced in Xcc by resuspension in 0.85% NaCl containing 50 μM Cu^2+^, and this was considered a good starting point for the experiments investigating the effect of environmental stress. Expression analysis using qPCR revealed that the expression of *relA* and *spoT* changed quite dramatically when log-phase cells of the wild-type Xcc strain 8004 (OD_600_ = 0.18) were exposed to the copper stress (Fig. 3). The relative expression of *relA* was most affected, rising dramatically in the first 12 h before falling slightly at 24 h and then increasing steadily over the next 10 days (Fig. 3A). In contrast, the relative expression of *spoT* initially decreased to a low of 1.33 ± 0.31 at 24 h before increasing slightly at 48 h and exhibited thereafter a very slight but continued increase in expression over the remaining 10 days (Fig. 3B). Despite these variations, it was interesting to note that the expression of *relA* and *spoT* was upregulated compared to that at 0 min under copper treatment throughout the entirety of the experiment according to the principle of 2^−ΔΔCT^ method (40).

**FIG 4:**
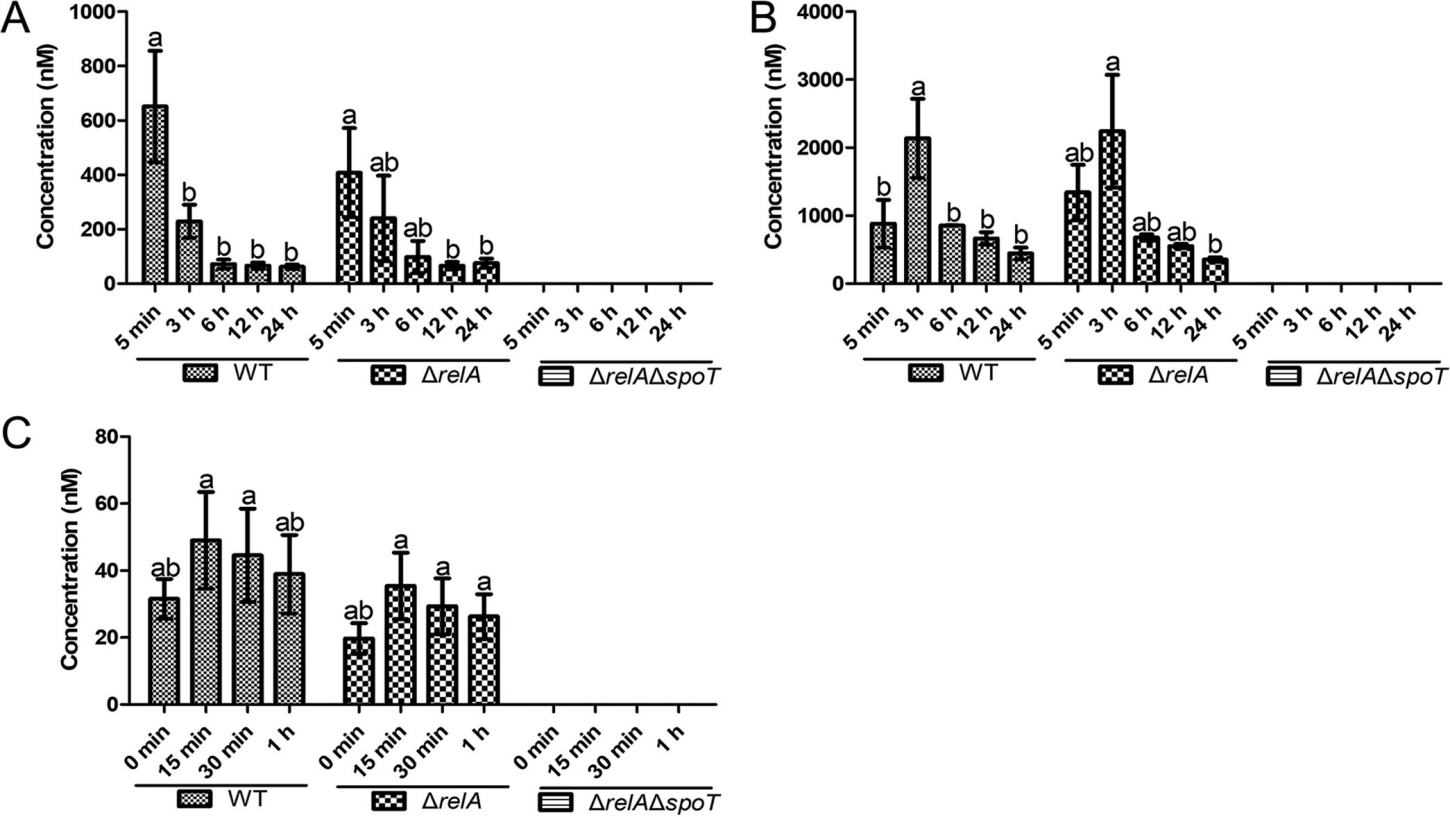
Level of ppGpp accumulation in the wild type and in Δ*relA* and Δ*relA*Δ*spoT* deletion mutants of Xanthomonas campestris pv. *campestris* exposed to abiotic stress. The concentration of ppGpp in the wild type (WT) and in Δ*relA* and Δ*relA*Δ*spoT* deletion mutants of Xcc was recorded at several time points after exposure to various abiotic stresses, including (A) copper stress induced by resuspension in 0.85% NaCl amended with 50 μM CuSO_4_ (5 min, 3 h, 6 h, 12 h, and 24 h), (B) oligotrophic stress caused by resuspension in 0.85% NaCl (5 min, 3 h, 6 h, 12 h, and 24 h), and (C) amino acid starvation induced by serine hydroxamate (SHX) treatment (0 min, 15 min, 30 min, and 1 h). Bars indicate one standard deviation from the mean (*n* = 3), while different letters above columns indicate significant differences (*P < *0.05) among samples at different time points in each strain according to a one-way ANOVA in conjunction with the least significant difference (LSD) test.

Similar experiments were designed to investigate the accumulation of ppGpp in the deletion mutants as well as in the wild type in response to similar stress. It was found that exposure to copper resulted in a rapid decline in ppGpp in both the wild type and the Δ*relA* deletion mutant to a low of approximately 100 nM within 6 h (Fig. 4A). However, the Δ*relA*Δ*spoT* double mutant completely lacked the capacity to produce ppGpp (Fig. 4). A similar pattern was also observed in response to the oligotrophic conditions imposed by the 0.85% NaCl treatment, but in this case, there was an initial increase in ppGpp at the 3 h time point and a more gradual decline thereafter (Fig. 4B). Comparable results were also observed in response to amino acid starvation induced by the serine hydroxamate (SHX), with the level of ppGpp slightly increasing at the 15 min time point in both the wild type and the Δ*relA* mutant before gradually falling over the next 45 min (Fig. 4C). However, the absolute level of ppGpp was significantly reduced compared to that of both 0.85% NaCl and 0.85% NaCl amended with 50 μM CuSO_4_ treatments. The fact that the Δ*relA*Δ*spoT* double mutant produced no discernible levels of ppGpp in response to SHX or, indeed, to any of the treatments throughout the experiment confirmed that *relA* and *spoT* were the sole genes responsible for ppGpp production in Xcc.

### The effect of RelA, SpoT, and ppGpp on induction of the VBNC state.

**FIG 5:**
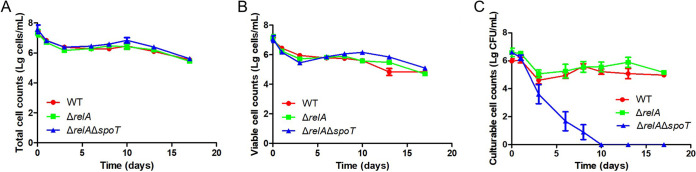
Effect of RelA and SpoT on induction of the VBNC state by oligotrophic condition in Xanthomonas campestris pv. *campestris*. The total number of cells (A), number of viable cells (B), and number of culturable cells (C) were recorded at various time points (0, 1, 3, 6, 8, 10, 13, and 17 d) after treatment with 0.85% NaCl to simulate oligotrophic condition, using a combination of flow cytometry and plating assays. WT, Δ*relA*, and Δ*relA*Δ*spoT* indicate the wild type, single mutant, and double mutant, respectively. Bars correspond to one standard deviation (SD) from the mean (*n* = 3).

Having established that both *relA* and *spoT* expression and ppGpp accumulation could have an important role in the stress response of Xcc and that the Δ*relA*Δ*spoT* double mutant completely lacked ppGpp, a further experiment was conducted to investigate whether the mutant strains differed from the wild type when being induced into the VBNC state. Little difference was found between the wild type and the Δ*relA* single mutant, but it was noted that most cells of the Δ*relA*Δ*spoT* double mutant could be induced into the VBNC state in response to the oligotrophic conditions resulting from resuspension in 0.85% NaCl (Fig. 5). The treatment resulted in a complete loss of culturability of the Δ*relA*Δ*spoT* double mutant by 10 d, while the wild type and the Δ*relA* single mutant seemed completely unaffected, even at the 17 d time point.

## DISCUSSION

The initial bioinformatic analysis performed in the current study confirmed that Xanthomonas campestris pv. *campestris* 8004 possessed homologues of both the *relA* and *spoT* genes. The predicted amino acid sequences were similar to those of other bacteria, such as E. coli, and contained all the characteristic functional domains commonly associated with the RelA/SpoT superfamily (Fig. S1). However, no evidence of any additional SASs was detected (Table S1). Single (Δ*relA*) and double (Δ*relA*Δ*spoT*) mutants were successfully generated using the triparental method, but it was not possible to produce a single gene Δ*spoT* mutant. Similar difficulty in generating a Δ*spoT* mutant has also been observed in a previous study of E. coli, a phenomenon the authors attributed to the essential hydrolyze activity of SpoT, which prevents ppGpp from accumulating to lethal levels in the bacterial cell (17). As expected, the deletion mutants from the current study exhibited many altered phenotypes, especially in the case of the Δ*relA*Δ*spoT* double mutant, which produced significantly lower levels of EPS and secreted enzymes and had a reduced capacity for biofilm formation and swarming motility. In general, most of these changes could be rescued by the reintroduction of a functional *relA* or *spoT* gene in the complementation strains, except the restoration of biofilm formation (Fig. 1D). A possible explanation might be that the complementation strain Δ*relA*(*relA*) was constructed using a shuttle plasmid that could not reintroduce the *relA* gene *in situ* and that it therefore lacked some of the surrounding regulatory elements, leading to unpredictable changes in gene expression. It was interesting to note that the reintroduction of *spoT* had a much more significant impact on phenotypes that were affected by both genes, with Δ*relA*Δ*spoT*(*spoT*) exhibiting a higher degree of phenotype recovery than Δ*relA*Δ*spoT*(*relA*) with regard to the secretion of extracellular enzymes, biofilm formation, and swarming motility, as well as pathogenicity (Fig. 1 and 2). It was also noted that in some cases, the loss of *relA* had no apparent effect on phenotype, for example, the secretion of extracellular enzymes or indeed pathogenicity (Fig. 1E to J and Fig. 2). Similar results have also been documented in previous studies of E. coli, where biofilm formation was significantly inhibited in the Δ*relA*Δ*spoT* mutant, while no significant difference was found between the Δ*relA* mutant and the wild type (41). The fact that the deletion of *relA* sometimes caused no change in a particular phenotype in the current study, and that *spoT* had a greater capacity to rescue phenotype in the Δ*relA*Δ*spoT* double mutant, provides strong evidence that ppGpp levels and downstream ppGpp-dependent phenotypes are predominantly mediated by SpoT in Xcc, while RelA has only a supplementary role.

Although Xcc is an important pathogen of crucifer crops, causing significant losses to global production, reports detailing pathogenicity factors in this species (42, 43) have given little attention to the stress responses of Xcc or the role of the key alarmone ppGpp during the infection process. In the genus *Xanthomonas*, the roles of DksA and (p)ppGpp in the virulence traits of *X. citri* subsp. *citri* were reported and the mechanism by RNA-seq was analyzed (11). There are no other publications focused on the ppGpp in the function of bacterial growth regulation and stress adaption in *Xanthomonas*. Here, the current study also revealed that ppGpp played a critical role in the pathogenicity of Xcc, with the ppGpp-deficient mutant Δ*relA*Δ*spoT* completely lacking the capacity to infect a viable host. Similar results have also been observed in other plant-pathogenic bacteria, such as *Ps. syringae* and *Er. amylovora*, as well as some human pathogens, including, Salmonella enterica serovar Typhimurium and V. cholerae (7, 9, 44, 45). The loss of pathogenicity observed in the current study is perhaps not surprising, as the ppGpp-deficient double mutant had reduced levels of EPS, cell wall degrading enzymes, biofilm formation, and swarming motility, all of which have previously been associated with pathogenicity in Xcc (46, 47) and are known to be associated with ppGpp in other plant-pathogenic species (8, 9). Here, the ppGpp-deficient mutant Δ*relA*Δ*spoT* reduced biofilm formation and swarming motility, which was consistent with the result of EPS production (Fig. 1). In bacterial global regulation, the EPS and flagella are part of the biofilm matrix, which is required for swarming motility (48–50). Meanwhile, ppGpp has also been associated with biofilm formation, as the regulation of quorum sensing (QS) can stimulate the synthesis of potential elements of the biofilm during the bacterial stringent response in bacteria (13). More specifically, ppGpp-deficient mutants of the human pathogen Listeria monocytogenes had a reduced capacity to adhere to polystyrene, which was attributed to reduced biofilm formation (51), while the upregulation of *spoT* in Helicobacter pylori led to the upregulation of an efflux pump that is known to be highly expressed during biofilm formation in this species (52). It is also possible that the increased sensitivity of the ppGpp-deficient Xcc mutant to environmental stress could have contributed to its reduced pathogenicity, as it has previously been suggested that the ppGpp-mediated stringent response might affect the survival of bacteria and limit their growth *in planta* (9). Meanwhile, there is also evidence that ppGpp has a regulatory role in the expression of specific pathogenicity factors in human pathogen V. cholerae and Salmonella enterica serovar Typhimurium (45, 53). Similarly, ppGpp has also been associated with the expression of genes associated with histidine metabolism, the type 3 secretion system (T3SS), the type 2 secretion system (T2SS), and TonB-dependent transporters in E. amylovora and *X. citri* subsp. *citri* (7, 11). Further research is therefore required to determine whether similar biological processes are also mediated by ppGpp in Xcc.

**FIG 6:**
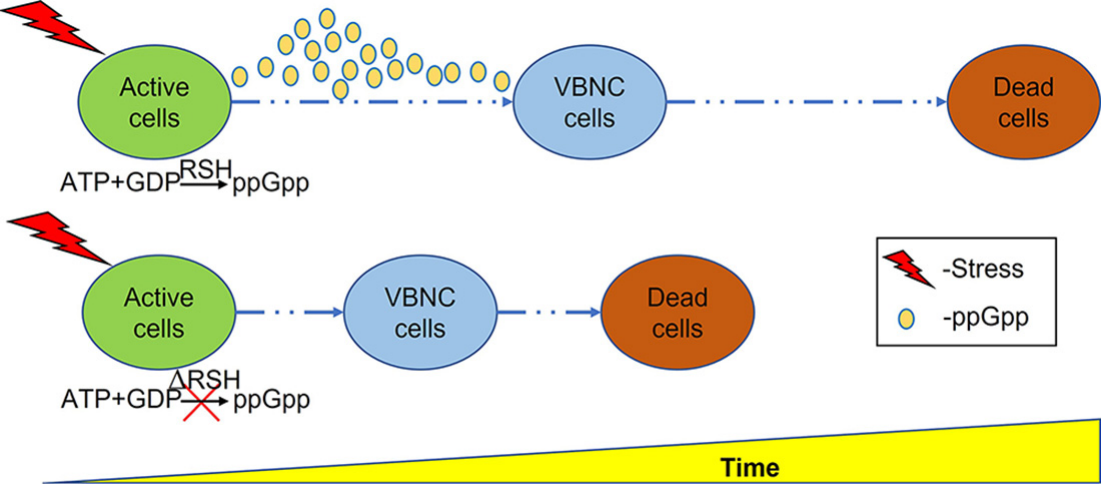
Schematic of the relationship between ppGpp and bacterial survival in response to environmental stress. In the wild type, ppGpp levels initially rise via the activity of the long RelA/SpoT homologue (RSH) proteins to trigger the stringent response under acute stress. However, in the ΔRSH (ppGpp-deficient) mutant, exposure to stress causes immediate induction into the VBNC, which cannot be sustained in the complete absence of ppGpp, ultimately resulting in premature cell death.

The current study also found that ppGpp had an important role during the response of Xcc to environmental stress. For example, it was found that the Δ*relA*Δ*spoT* double mutant was almost completely incapable of growth on minimal medium (Fig. 1A) and that ppGpp levels were correlated with exposure to stress, rising shortly after exposure but then falling off quite rapidly (Fig. 4), which could suggest the triggering of a short-term stringent response, as increased ppGpp levels have previously been associated with the reallocation of cellular resources to facilitate survival in *Ps. syringae* (15) and the growth of E. coli in response to nutrient limitation (54). Moreover, it can be inferred that ppGpp could regulate the gene expression of flagellum to control motility, because ppGpp deficiency significantly reduced swarming ability in the Δ*relA*Δ*spoT* of X. campestris pv. *campestris* (Fig. 1K and L). As is well known, the flagellum plays an important role in chemotaxis to help bacteria to find optimal conditions for survival (55). In addition, when environmental fluctuations occur, ppGpp can be synthesized by RSH enzymes which monitor key metabolites (amino acid, fatty acids, or tricarboxylic acid [TCA] intermediates) to resist the stress (56). The accumulation of ppGpp has also been associated with the upregulation of stress-related genes in E. coli, as well as altered transcription and translation to maintain cellular homeostasis under adverse conditions (57–61). Similarly, ppGpp has been shown to affect enzymes related to nucleotide synthesis and nucleotide metabolism (especially the cellular level of GTP), which can affect key cellular processes associated with the survival of bacteria (6, 62–66). Therefore, cellular metabolites, especially GTP level, and stress-related enzymes should be further studied in Xcc to allow us to understand the ppGpp-related regulation mechanism. Given the extent of the biological processes mediated by ppGpp, it is perhaps not surprising that the ppGpp-deficient Xcc mutant investigated in the current study was found to have increased sensitivity to environmental stress and greater susceptibility to entering the VBNC state in response to oligotrophic conditions (Fig. 4 and 5), results that were consistent with a previous study that reported that although ppGpp-deficient mutants of E. coli lost culturability earlier than the wild type, they were also less effective in maintaining the VBNC state (38). Taken together, these results also provide clues as to the mechanism triggering the stress response in Xcc, where exposure to acute stress causes a peak in ppGpp that triggers a stringent response, while sustained exposure causes ppGpp levels to decrease. The ppGpp-deficient mutant can enter into VBNC state earlier and maintain this period for a very short time, ultimately resulting in premature cell death. This hypothesis, which has been illustrated in Fig. 6, provides a plausible explanation as to why the ppGpp-deficient mutant in the current study was found to survive for a short time and rapidly lose viability in response to environmental stress. In brief, this is the first study to explore the relationship between ppGpp and stress resistance, especially ppGpp and VBNC state, in *Xanthomonas*.

Although ppGpp is a highly conserved secondary messenger in bacteria, there is still much to be learned regarding the exact mechanism by which it influences cellular process. However, two different regulatory mechanisms have been described in response to different aspects of nutrient deficiency. In response to amino acid starvation, deacylated tRNA binds to the ribosomal A site, inducing ppGpp synthesis by RelA (5, 67), while in response to fatty acid starvation, a conformational change occurs in the acyl carrier protein (ACP), causing it to bind to SpoT and triggering the synthesis of ppGpp (4, 56, 68). The ppGpp-dependent VBNC/stringent response observed in Xcc exposed to copper in the current study is therefore of great interest, although further investigation is required to fully characterize the regulatory mechanism in detail.

In summary, the current study found strong evidence that ppGpp, and SpoT in particular, has a very important role in mediating both the stress response and pathogenicity of Xcc. A hypothetical model illustrating the shorter interval from viability to death through the VBNC state for ppGpp-deficient mutant under stress was developed, although the precise molecular mechanisms by which ppGpp exerts its influence remain to be characterized completely. Nonetheless, the findings of the current study have much advanced our understanding of the significance of ppGpp in the biology of Xcc and could have important implications for more effective management of this important pathogen.

## MATERIALS AND METHODS

### Bacterial strains, plasmids, and culture conditions.

The bacterial strains and plasmids used in the current study have been listed in Table 1. The parental isolate Xcc strain 8004 (69), as well as the derived mutants, was routinely cultured in LB broth (5 g/L yeast extract, 5 g/L NaCl, and 10 g/L tryptone) or NYG broth (3 g/L yeast extract, 5 g/L hipolypeptone, and 20 g/L glycerol) at 28°C with shaking (120 rpm) for 12 h, while the Escherichia coli isolates were cultured in LB broth at 37°C with shaking (200 rpm). When required, solid medium was prepared by the addition of 12.8 g/L agar, and selective antibiotics were added at the following concentrations: rifampicin, 50 μg/mL, kanamycin, 50 μg/mL, chloramphenicol, 20 μg/mL, and tetracycline, 5 μg/mL for Xcc and 15 μg/mL for E. coli.

**TABLE 1:** Bacterial strains and plasmids used in the current study^*a*^

Strains and plasmids	Properties	Source
Bacterial strains		
8004	Xanthomonas campestris pv. *campestris* wild-type isolate, Rif^R^	69
Δ*relA*	Xcc 8004 *relA* deletion mutant, Rif^R^	Current study
Δ*relA*Δ*spoT*	Xcc 8004 *relA* and *spoT* double deletion, ppGpp-deficient mutant, Rif^R^	Current study
Δ*relA*(*relA*)	Complementation isolate: Δ*relA* containing pLAFR3relA, Rif^R^, Tc^R^	Current study
Δ*relA*Δ*spoT*(*relA*)	Complementation isolate: Δ*relA*Δ*spoT* containing pLAFR3relA, Rif^R^, Tc^R^	Current study
Δ*relA*Δ*spoT*(*spoT*)	Complementation isolate: Δ*relA*Δ*spoT* containing pLAFR3spoT, Rif^R^, Tc^R^	Current study
pL3-WT	Control isolate: Xcc 8004 containing pLAFR3, Rif^R^, Tc^R^	Current study
pL3-Δ*relA*	Complementation control isolate: Δ*relA* containing pLAFR3, Rif^R^, Tc^R^	Current study
pL3-Δ*relA*Δ*spoT*	Complementation control isolate: Δ*relA*Δ*spoT* containing pLAFR3, Rif^R^, Tc^R^	Current study
DH5α	Escherichia coli, F-φ80d*lac*ZΔM15Δ(*lac*ZYA-*arg*F) U169*end*A1*rec*A1*hsd*R17(rk^–^,mk^+^)*sup*E44λ-*thi*-1 *gyr*A96 *relA1 phoA*	TransGen Biotech Company, Beijing, China
Plasmids		
p2P24Km	Suicide vector for in-frame deletion, derived from pEx18-KCL containing SacB selectable marker; Km^R^	70
p2P24Km*relA*	p2P24Km containing *relA* fragment, Km^R^	Current study
p2P24Km*spoT*	p2P24Km containing *spoT* fragment, Km^R^	Current study
pRK600	Helper plasmid for triparental mating ColE1 *oriV*; RP4; tra+; RP4 *oriT*; Cm^R^	71
pLAFR3	Xcc expression vector containing RK2 replicon, Tc^R^	73
pLAFR3*relA*	Complementation vector: pLAFR3 containing *relA* gene, Tc^R^	Current study
pLAFR3*spoT*	Complementation vector: pLAFR3 containing *spoT* gene, Tc^R^	Current study

a Rif^R^, Km^R^, Cm^R^, and Tc^R^ indicate resistance to rifampicin, kanamycin, chloramphenicol, and tetracycline, respectively.

### Bioinformatics analysis.

The putative Xcc RelA and SpoT sequences identified in a previous survey of the RelA/SpoT superfamily (16) were retrieved from the Xcc 8004 genome database (NCBI). Functional domains within the RelA and SpoT sequences were identified using the InterPro online tool (https://www.ebi.ac.uk/interpro/search/sequence/), and the amino acid sequences of both the RelA and SpoT synthetase domains were used as a query to search the Xcc 8004 genome for other homologous proteins.

### Construction of knockout mutants.

The Δ*relA* single mutant and Δ*relA*Δ*spoT* double mutant were generated from the parental Xcc 8004 strain using the triparental mating methods. Five hundred base pair upstream and downstream fragments of the Xcc *relA* and *spoT* genes were amplified using the XccRelAF8-F/R, XccRelAF10-F/R, XccSpoTF8-F/R, and XccSpoTF10-F/R primer sets, respectively, to produce amplification products that contained a fusion sequence compatible with the p2P24Km vector (70). The resulting PCR fragments were ligated by corresponding primers XccRelAF7B-F/XccRelAF9S-R and XccSpoTF7E-F/XccSpoTF9H-R using the In-Fusion method, and the resulting fusion products were cloned into the p2P24Km vector as restriction fragments (BamHI/SalI in the case of *relA* and EcoRI/HindIII for *spoT*) to yield the two donor vectors p2P24Km*relA* and p2P24Km*spoT*, respectively.

The triparental mating was conducted using E. coli donors containing either the p2P24Km*relA* or the p2P24Km*spoT* plasmid in conjunction with an E. coli helper strain containing pRK600 (71) to transform the Xcc 8004 (wild type [WT]) receptor. The mating itself involved mixing 300-μL aliquots of the three strains, which were collected in the log phase of growth (OD ≈ 1), to produce a 900 μL mixture that was subsequently harvested by centrifugation (12,000 rpm for 1 min) and washed twice in 300 μL sterilized water before finally being resuspended in 100 μL sterilized water and spread on an NYGA plate. After overnight incubation at 28°C, the bacterial mixture from each plate was collected in sterile water and used to prepare 10-fold serial dilutions that were plated on fresh NYGA plates containing rifampicin and kanamycin. After a further 2 days of incubation at 28°C, individual *Xanthomonas*-like colonies (yellow in color) were picked and verified by PCR using the Xcc-specific primers DLH120/125 detailed in a previous study (72) to confirm that the picked colonies were indeed Xcc. Further PCRs using XccrelA-F10/R10, XccrelA3-F/R, XccspoT-F/R, and XccspoT1-F/R primer sets were conducted to verify that the colonies had been successfully transformed. Colonies with the expected amplicon profile were then subjected to a second round of screening on NYGA containing 8% sucrose supplemented with rifampicin or both rifampicin and kanamycin. Transformants that were capable of growth in the presence of rifampicin but not kanamycin were then selected and reverified by PCR as described above. A similar process was used to generate the Δ*relA*Δ*spoT* double mutant, but in this case, the recipient strain was the Xcc Δ*relA* single mutant in conjunction with helper and donor E. coli strains containing pRK600 and p2P24Km*spoT*, respectively. All the primers used in the cloning and verification process are listed in Table S2.

### Mutant complementation.

Complementation strains reintroducing functional *relA* and *spoT* genes to the deletion mutants were also generated by triparental mating. In this case, the full-length genes including the native promoter were amplified using the XccrelAHB1E-F/1B-R and XccspoTHB2B-F/2H-R primer sets (Table S2) and cloned into the pLAFR3 (73) vector to yield the plasmids pLAFR3*relA* and pLAFR3*spoT*, respectively. The resulting E. coli transformants were then used as the donor strains in the protocol outlined above in conjunction with Δ*relA*, Δ*relA*Δ*spoT*, and wild-type (pLAFR3 control) Xcc receptor strains.

### RNA extraction and qPCR analysis.

The relative expression of *relA* and *spoT* was initially assessed in wild-type cells exposed to copper, which were prepared in a manner identical to that described in the ppGpp extraction experiment detailed below (OD_600_ = 2.0), and sampled at 6 time points (0 min, 5 min, 12 h, 1 d, 2 d, 10 d). A second expression experiment was then used to assess the expression of the two genes in the wild-type isolate, as well as the deletion mutants and complementation strains without copper treatment, during log-phase growth (OD_600_ = 1.0). In both cases, the cells were harvested by centrifugation at 12,000 rpm for 3 min at 4°C and total RNA was extracted using the SV total RNA isolation system (Promega Corporation, Beijing, China) according to the protocol of the manufacturer. The quality and quantity of the resulting RNA were determined using NanoDrop 2000 (Thermo Scientific, Beijing, China) before being stored at −80°C until required. First-strand cDNA was synthesized using the PrimeScript RT reagent kit with gDNA Eraser (TaKaRa, Beijing, China) and stored at –20°C.

The relative expression of the Xcc *relA* and *spoT* genes was determined by quantitative PCR (qPCR), which was performed using the Applied Biosystems 7500 fast real-time PCR system (Life Technologies, USA) in conjunction with SYBR Premix DimerEraser (TaKaRa, Japan). Each reaction mixture (20 μL) contained 2 μL diluted cDNA, 10 μL SYBR Premix DimerEraser, each primer at a final concentration of 500 nM, 0.4 μL Rox Reference Dye II, and 5.6 μL of sterile distilled water and processed using the following PCR program: 95°C for 30 s, followed by 40 cycles of 95°C for 5 s, 56°C for 30 s, and 72°C for 30 s. The standard curve was generated using 4-fold dilutions of the template cDNA, and the PCR efficiency (E) was calculated using a linear regression model: E (%) = (10^−1/slope^ − 1) × 100% (74). The 2^−ΔΔCT^ method was used to calculate the relative expression level of the target genes (40), and the *Ct* (cycle threshold) values of each gene were normalized to *Ct* values of the housekeeping genes *ugpC* and *pbpA*. Each treatment was represented by three biological replicates and the entire experiment was conducted three times. All the primers used for qPCR analysis are listed in Table S3.

### Growth, EPS, biofilm, extracellular enzymes, and swarming motility assays.

The growth of the deletion mutants, complementation strains, and wild-type 8004 Xcc strain was evaluated on three types of solid media, including LBA and NYGA as well as a modified minimal medium (mMM) containing 40 mmol/L MOPS (morpholinepropanesulfonic acid) and 4 mmol/L tricine (adjusted to pH 7.2 with KOH), 50 mmol/L KCl, 10 mmol/L NH_4_Cl, 0.5 mmol/L MgSO_4_ · 7H_2_O, 0.4% glucose, and 1.6% agar, which was included to investigate the effect of oligotrophic stress (75). Each plate was inoculated with 10-fold serial dilutions of a stock bacterial suspension adjusted to an OD_600_ of 0.3. The resulting colonies were counted after 4 days of incubation at 28°C in the case of LBA and NYGA and after 5 days in the case of mMM.

The production of EPS was investigated using NYGA amended with 2% glucose, as described in a previous study (76). Two-microliter aliquots of each Xcc suspension (OD_600_ = 0.3) were carefully pipetted onto separate plates and cultured at 28°C for 4 days before the size and morphology of the resulting colonies were assessed.

Similar plate-based assays were also used to assess the enzyme activity of the different Xcc strains. In this case, NYGA amended with 1% (wt/vol) skimmed milk, 0.1 (wt/vol) soluble starch, or 0.5% (wt/vol) carboxymethyl cellulose was used to estimate the extracellular protease, amylase, and cellulase activities, respectively, in accordance with the protocols of previous studies (77–79). Again, 2-μL aliquots of bacterial suspension (OD_600_ = 0.3) were inoculated onto each plate, and the degree of enzyme activity was quantified by measuring the diameter of transparency (zone of activity) around each colony after 24 h of incubation at 28°C.

The biofilm assay was conducted using 9-mL liquid cultures (LB) inoculated with 1-mL bacterial suspensions (OD_600_ = 1). After 5 days of incubation at 28°C, the bacterial cultures were discarded from the tubes, replaced with an equivalent volume (10 mL) of 0.1% (wt/vol) crystal violet (CV), and allowed to stain for 30 min. The CV was then removed, and the tube was carefully washed twice with sterilized water before the pigment bound to the inside of the tube was eluted in absolute ethanol and quantified by spectrophotometry at 630 nm in accordance with the protocol of a previous study (80).

The swarming motility of the different Xcc strains was assessed using the plate assay described in a previous study (81). In this case, 2-μL aliquots of each bacterial suspension (OD_600_ = 0.3) were inoculated onto semisolid NYGA plates (3 g/L Noble agar), and the diameters of the resulting growth zones were measured after 4 days of incubation at 28°C in order to calculate the total colony area.

In all cases, each treatment was represented by three biological replicates, and the entire experiment was conducted three times.

### Pathogenicity assay.

The pathogenicity of the deletion mutants, complementation strains, and wild-type Xcc 8004 strain were assessed on radish (Raphanus sativus L. cv. Japan 501) leaves. Twenty microliters of the inoculum, which was prepared from overnight cultures by centrifugation at 12,000 rpm for 3 min and resuspension in 0.85% NaCl (OD_600_ = 0.3), was injected into the main vein on the back of randomly selected leaves of radish seedlings at the 5-true-leaf stage. Identical negative-control samples were prepared by injecting an equivalent volume of sterile 0.85% NaCl. Symptoms of disease were observed and evaluated at 7 days postinoculation (dpi), when 3 leaf disks (9 mm) were collected from each inoculated leaf using a hole puncher and processed for bacterial isolation and counting. The leaf disks were first disinfected by immersion in a 1% NaOCl solution for 1 min before being washed twice in sterilized water and frozen in liquid nitrogen. The samples were then homogenized using a Retsch MM400 ball-milling machine at 60 Hz for 1 min. The resulting leaf powder was resuspended in 0.85% NaCl and a 10-fold dilution series plated on LBA. The number of resulting colonies was counted after 2 days of incubation at 28°C. Each treatment was represented by 6 individual seedlings, and the entire experiment was conducted three times.

### Extraction and measurement of ppGpp during exposure to environmental stress.

The accumulation of ppGpp in response to environmental stress (copper exposure, oligotrophic conditions, and amino acid starvation) was assessed in both the wild type and the deletion mutants. In preparation, each strain was cultured on solid medium (LBA) for 48 h at 28°C, before a single colony was picked and inoculated into 10 mL LB broth and incubated at 28°C with shaking (120 rpm) for 12 h, after which the cells were harvested by centrifugation.

In the case of the copper and oligotrophic stress, the cells were resuspended to an OD_600_ of 0.18 in either 0.85% (wt/vol) NaCl solution amended with CuSO_4_ to a final concentration of 50 μM or 0.85% (wt/vol) NaCl alone. The cells were then kept at 28°C and samples were taken at intervals (5 min, 3 h, 6 h, 12 h, and 24 h) for ppGpp extraction as described below.

The cells in the amino acid starvation treatment were washed in LB broth and then resuspended in M9 broth (11.28 g/L 5×M9 minimal salts, 0.1 M MgSO_4_ · 7H_2_O, 0.1 M CaCl_2_, and 15 g/L glucose; OD_600_ = 2) amended with 0.8 g/L serine hydroxamate (SHX) to induce amino acid starvation (82). The cells were kept at 28°C and ppGpp was extracted at three time points (15 min, 30 min, and 1 h), with time zero being represented by measurements taken before the addition of SHX.

The ppGpp extraction itself was conducted using a cold methanol method developed in a previous study (83). The bacterial cells were harvested by centrifugation at 4,000 rpm for 10 min at 4°C and resuspended in 2 mL of ice-cold 100% methanol. The samples were then vortexed for 50 s before being frozen in liquid nitrogen and allowed to thaw on ice. Each sample was centrifuged a second time at 4,000 rpm for 10 min, and the supernatant was collected and kept on ice, while the pellet was subjected to a second round of extraction. The supernatants from both extractions were then combined and filtered through 0.22-μm filter paper. The filtrate was dried by centrifugal drying, resuspended in 200 μL distilled water, and kept at −20°C until required for the liquid chromatography-tandem mass spectrometry (LC-MS/MS) analysis, which was performed using the Agilent 1290 liquid chromatography system linked to a 6460 triple quad mass spectrometer (Agilent, USA). The LC separation itself was performed using an ACQUITY UPLC HSS T3 column (2.1 mm by 50 mm, 1.8 μm, Waters, Milford, MA, USA), with methanol as mobile phase A and 10 mM ammonium acetate, 0.02% ammonium hydroxide in water as mobile phase B, with the linear gradient program set as follows: 0 to 3 min, 0% A; 3 to 5 min, 0% A to 90% A; 5 to 6 min, 90% A; 6 to 6.1 min, 90% A to 0% A; 6.1 to 10 min, 0% A. The flow rate was set at 0.2 mL/min, with an injection volume of 10 μL. The mass spectra were acquired under positive electrospray ionization, and the ppGpp concentration was detected according to its characteristic ion peak (*m/z* 604→*m/z* 152) (9) in comparison to standardized ppGpp stock solutions (TriLink Biosciences Company, San Diego, CA, USA). Each treatment was represented by a single biological replicate, and the entire experiment was conducted three times.

### Determination of viable and culturable cells in response to oligotrophic condition.

The cells used to assess the effect of oligotrophic condition (0.85% NaCl) on the induction of the VBNC state were prepared in a manner identical to that described above.

The flow cytometry (FCM), itself, was performed using the FACSCalibur system (BD Biosciences, CA, USA) in conjunction with SYTO 9 (Invitrogen) and propidium iodide (PI; Invitrogen) to measure the total and viable population of Xcc cells in the induction mixture, respectively, as described in a previous study (29). Each treatment was separated into two samples, which were stained with either SYTO 9 (final concentration 4 μM) or PI (final concentration 30 μM) for 30 min at room temperature before 800-μL aliquots were transferred to trucount tubes (BD Biosciences) for green and red fluorescence analysis, respectively. The number of viable cells was then estimated by subtracting the number of PI-stained cells from the number of SYTO 9-stained cells (total number of cells), while the number of culturable cells was measured using the conventional plate counting method (29). Each treatment was represented by two biological replicates, and the entire experiment was conducted three times.

### Statistical analysis.

The enzyme activity, biofilm formation, swarming motility, and pathogenicity data collected in the current study were subjected to a one-way analysis of variance (ANOVA) using GraphPad Prism 5 software with significant differences (*P < *0.05) between treatments being determined by Dunnett’s multiple-comparison test. However, the cell counts on the media (NYGA, mMMA, and LBA) were assessed using a two-way ANOVA (GraphPad Prism 5) in comparison to the wild-type sample, while the ppGpp accumulation data were analyzed by a one-way ANOVA (DPS software) with statistical differences (*P < *0.05) determined by the least significant difference (LSD) test.

### Data availability.

The data and material can be available from the authors.
