# Supplementary material for: The Role of RelA and SpoT on ppGpp Production, Stress Response, Growth Regulation, and Pathogenicity in Xanthomonas campestris pv. campestris
Source: Microbiol Spectr. 2021 Dec 22;9(3):e02057-21. doi: 10.1128/spectrum.02057-21 (PMC8693919; doi:10.1128/spectrum.02057-21)
Supplement: SUPPLEMENTAL FILE 1 — Supplemental material. Download SPECTRUM02057-21_Supp_1_seq9.pdf, PDF file, 0.36 MB [file spectrum02057-21_supp_1_seq9.pdf]

Supplemental data

Table S1 Proteins exhibiting homology with the RSH ppGpp synthetase domains in *Xanthomonas campestris* pv. *campestris* 8004

Table S2 Primers used for the construction and verification of the Δ*relA* and Δ*relA*Δ*spoT* mutants and complementation strains

Underlined nucleotides indicate the location of restriction sites used for cloning

Table S3 Primers used for qPCR analysis in the current study

Figure S1 Predicted domains within the RelA and SpoT homologues in *Xanthomonas campestris* pv. *campestris*. Bioinformatic analysis using the InterPro online tool indicated that both proteins contained the ppGpp synthetase domain as well as other characteristic motifs of long RelA/SpoT homologue (RSH) proteins: HD, hydrolysis domain; SYNTH, synthesis domain; TGS, ThrRS (threonyl tRNA synthetase), GTPase, and SpoT; ACT, aspartokinase, chorismate mutase, and TyrA (prephenate dehydrogenase). The terms NTD and CTD indicate the amino-terminal domain and carboxy-terminal domain, respectively, while numbers indicate the amino acid location within the protein.

Figure S2 Gene expression of *relA* and *spoT* in *Xanthomonas campestris* pv. *campestris* deletion mutants and complementation strains during log-phase growth. The bar charts indicate the *relA* (A) and *spoT* (B) relative expression of mutant strains in comparation to wild-type strain. WT, Δ*relA*, and Δ*relA*Δ*spoT* indicate the wild-type, single mutant and double mutant, respectively, while the prefix pL3- indicates control strains used during complementation, and the suffixes (*relA*) and (*spoT*) indicate complementation with functional copies of *relA* and *spoT*, respectively. Bars correspond to one standard deviation (SD) from the mean (n = 3).

Table S1 Proteins exhibiting homology with the RSH ppGpp synthetase domains in *Xanthomonas campestris* pv. *campestris* 8004

Query sequence	Gene ID (NCBI)	Sequence identity (%)	Functional annotation	P value
RelA ppGpp synthetase domain	XC_RS05900 (*relA*)	100	Bifunctional (p)ppGpp	7.00E-92
	XC_RS04795 (*spoT*)	52.71	Synthetase/guanosine-3',5'-bis(diphosphate)	6.00E-36
	XC_RS07530	28.26	Ribonuclease G	2
	XC_RS15935	29.55	EAL domain-containing protein	2.6
	XC_RS08665	24.53	TonB-dependent receptor	4.9
SpoT ppGpp synthetase domain	XC_RS04795 (*spoT*)	100	Synthetase/guanosine-3',5'-bis(diphosphate)	6.00E-88
	XC_RS05900 (*relA*)	53.6	Bifunctional (p)ppGpp	2.00E-35
	XC_RS21215	45.45	Alpha-glucuronidase	2
	XC_RS04155	32.65	Membrane protein	3.2
	XC_RS00865	42.31	Amino acid ABC transporter permease	3.5
	XC_RS16350	34.69	Recombination protein RecR	3.5

Table S2 Primers used for the construction and verification of the Δ*relA* and Δ*relA*Δ*spoT* mutants and complementation strains

Target gene	Primer	Sequence (5'→3')	Restriction site	Product size (bp)	Annealing temperature (°C)
hrpF	DLH120	CCGTAGCACTTAGTGCAATG		619	60
	DLH125	GCATTTCCATCGGTCACGATTG			
spoT	XccSpoTF8-F	ATGACCATGATTACGACACTCCGCCCCCTTTCC		500	70
	XccSpoTF8-R	CAGTGATGCGCGCGGGCCGGAAGACTATGCCAG			
	XccSpoTF10-F	CCGCGCGCATCACTGCGGCGCATGTCGGCGCAG		500	70
	XccSpoTF10-R	TGCATGCCTGCAGGTGACCAGCAACCGCTGGTC			
	XccSpoTF7E-F	CGGAATTCACACTCCGCCCCCTTTCC	*Eco*RI	1000	70
	XccSpoTF9H-R	CCAAGCTTGACCAGCAACCGCTGGTC	*Hin*dIII		
	XccspoT-F	TCTGCAACTCAAGGAGATGGTC		3425	60
	XccspoT-R	GGCTGATTCCAAAGGAAACTGA			
	XccspoT1-F	AAATTCCGACGCCAGTTCTTC		142	56
	XccspoT1-R	CGAGCCCTACATCACCCATCC			
	XccspoTHB2B-F	CGGGATCCTCCAAAGGAAACTGAATAAGCG	*Bam*HI	2747	62
	XccspoTHB2H-R	CCAAGCTTACATGCGCCGCAGTGAT	*Hin*dIII		
relA	XccRelAF8-F	AGCTCGGTACCCGGGGTGGCAATTCCACTGCGG		500	70
	XccRelAF8-R	GAAGACTGCGTGCGCAAAGGCGGCAGGGACATG			
	XccRelAF10-F	GCGCACGCAGTCTTCGCAGCCGGGGCGGCTAC		500	70
	XccRelAF10-R	ATGCCTGCAGGTCGACTGCGCCTGCGCCACTTC			
	XccRelAF7B-F	CGGGATCCGTGGCAATTCCACTGCGG	*Bam*HI	1000	70
	XccRelAF9S-R	GCGTCGACCTGCGCCTGCGCCACTTC	*Sal*I		
	XccrelA-F10	TCCACGTACTGCGGATTGAGC		3221	56
	XccrelA-R10	CGCCGGAACACGATGACACT			

	XccrelA3-F	CCGCGATGTGGACGAAACC		142	56
	XccrelA3-R	CTTCCGCCAGATGCTGTAGATG			
	XccrelAHB1E-F	CGGAATTCCACCGTCCGCTTGCTGC	*Eco*RI	2800	68
	XccrelAHB1B-R	CGGGATCCATGCGGTGGCGTCCGTC	*Bam*HI		
pLAFR3	pR3-conF1	TGCCGTGCTCGTGTTCGGGGG		~300+insert	58
	pR3-conR1	GAGTTAGCTCACTCATTAGG			

Underlined nucleotides indicate the location of restriction sites used for cloning

Table S3 Primers used for qPCR analysis in the current study

Target gene	Primer	Sequence (5'→3')	Final Conc. (nM)	Product size (bp)	R^2	Efficiency (%)
pbpA	*pbpA*-F	GACGGGCCACTCCACTTCTG	500	199	0.9995	95
	pbpA-R	GCAACAACGGCGTGCTCAAC				
ugpC	*ugpC*-F	AAGGTGTCCGCAAGGTCTACGA	500	159	0.9983	103
	ugpC-R	TGCCCGCACTGATGTCCTCC				
relA	Xcc*relA*3-F	CCGCGATGTGGACGAAACC	500	142	0.9996	100
	Xcc*relA*3-R	CTTCCGCCAGATGCTGTAGATG				
spoT	Xcc*spoT*1-F	AAATTCCGACGCCAGTTCTTC	500	142	0.9955	93
	Xcc*spoT*1-R	CGAGCCCTACATCACCCATCC				

Figure S1

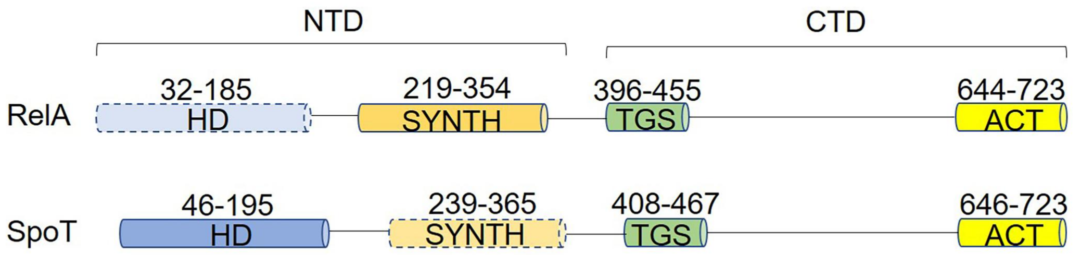

Figure S1 Predicted domains within the RelA and SpoT homologues in *Xanthomonas campestris* pv. *campestris*. Bioinformatic analysis using the InterPro online tool indicated that both proteins contained the ppGpp synthetase domain as well as other characteristic motifs of long RelA/SpoT homologue (RSH) proteins: HD, hydrolysis domain; SYNTH, synthesis domain; TGS, ThrRS (threonyl tRNA synthetase), GTPase, and SpoT; ACT, aspartokinase, chorismate mutase, and TyrA (prephenate dehydrogenase). The terms NTD and CTD indicate the amino-terminal domain and carboxy-terminal domain, respectively, while numbers indicate the amino acid location within the protein.

Figure S2

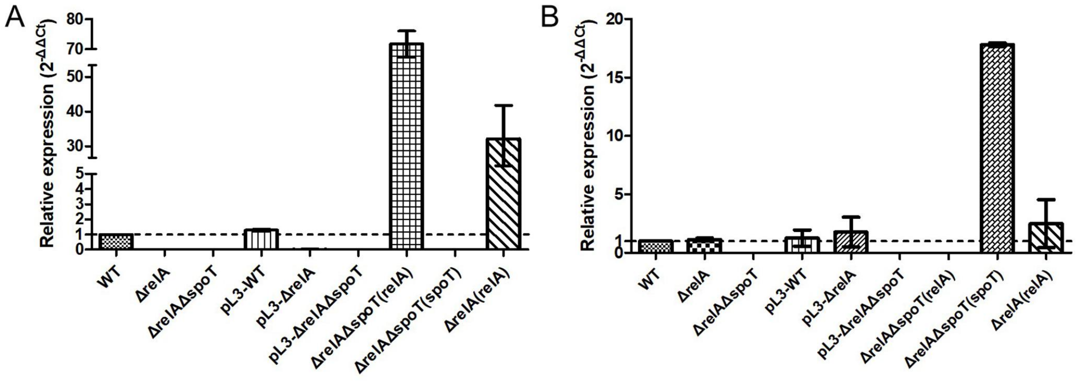

Figure S2 Gene expression of *relA* and *spoT* in *Xanthomonas campestris* pv. *campestris* deletion mutants and complementation strains during log-phase growth. The bar charts indicate the *relA* (A) and *spoT* (B) relative expression of mutant strains in comparation to wild-type strain. WT, Δ*relA*, and Δ*relA*Δ*spoT* indicate the wild-type, single mutant and double mutant, respectively, while the prefix pL3- indicates control strains used during complementation, and the suffixes (*relA*) and (*spoT*) indicate complementation with functional copies of *relA* and *spoT*, respectively. Bars correspond to one standard deviation (SD) from the mean (n = 3).
